# Pickering Emulsions as Catalytic Systems in Food Applications

**DOI:** 10.1021/acsfoodscitech.4c00839

**Published:** 2025-01-09

**Authors:** Joan Oñate Narciso, Robert Soliva-Fortuny, Laura Salvía-Trujillo, Olga Martín-Belloso

**Affiliations:** † Department of Food Technology, Engineering and Science, Universitat de Lleida − Agrotecnio CeRCA Center, Avda. Alcalde Rovira Roure 191, 25198 Lleida, Spain

**Keywords:** Pickering-assisted catalysis, Pickering interfacial catalysis, Pickering emulsions, enzymes, catalysis, food systems

## Abstract

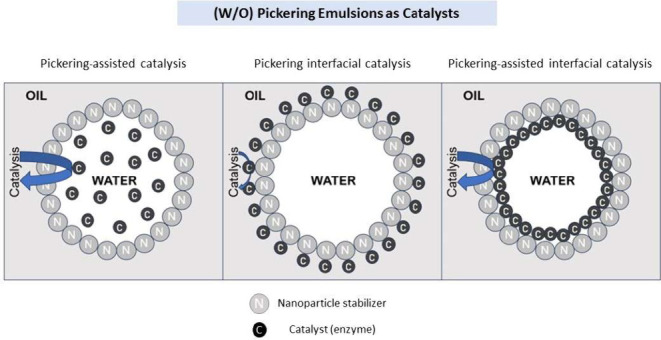

Enzyme catalysis is important in food processing, such as in baking, dairy, and fiber processing and beverages. A recent advancement in catalysis is the development of Pickering emulsions as enzymatic catalytic systems; however, the use of Pickering emulsions (PEs) as catalytic systems in foods remains largely underdeveloped. Challenges exist that inhibit the widespread adoption of PEs as catalytic systems in foods. These limitations include the limited food-grade solid particle stabilizers, their poor dual wettability, and the potential effects of surface modifications of the solid particles on the stability and efficiency of the PEs. In this Review, the two types of PE catalysis (Pickering-assisted catalysis and Pickering interfacial catalysis), their formation, and some of their applications in the food industry are presented. In addition, the proposed solutions and strategies to improve the PE catalyst design are introduced. An outlook on how the field of PE catalysis will progress is briefly highlighted.

## I Introduction

Pickering emulsions (PEs) were first described by Ramsden in 1903 and only in 1907 by Pickering.^1,2^ PEs are composed of immiscible fluids, usually oil and water, stabilized by amphiphilic organic or inorganic solid particles. Inorganic solid particles can be rendered amphiphilic by *in situ* hydrophobization, such as by using minimal amounts of cationic surfactants^3,4^ The stabilizing effect of the solid particles renders PEs to be generally more stable than emulsions obtained with a surfactant. The adsorption free energy, which is the energy required to remove a solid particle from the interface of the emulsion, is much higher in PEs than in conventional emulsions.^5^ Because of the high energy required to remove the particles, the adsorbed solid particles in the O/W interface of PEs are prevented from spontaneous detachment due to their own Brownian motion. The particles can act as a space barrier between the immiscible fluids to prevent the droplets from coalescing.^6^ PEs also have the advantages of reduced production cost, low toxicity, and favorable biocompatibility.^7^ When PEs are formed, the particles should be fractionally wetted by both oil and water phases so that the solid particles will be adsorbed at the interface. PE formation and stability depend on factors such as wettability of the particles, their concentration, size, density, and packing configuration.^8^ Oil-in-water (O/W) emulsions are formed when the particles have a contact angle lower than 90° (that is, the particles are hydrophilic) and water-in-oil (W/O) emulsions are formed with hydrophobic particles with a contact angle greater than 90°.^9^ These PEs are stabilized by the formation of a three-dimensional network between the particles and the two phases.

Nanoparticle-stabilized PEs are remarkably stable. A few examples of solid particles used in PEs are metal oxide and hydroxide nanocomposites^10,11^ and, in the case of food-grade stabilizers, starch nanoparticles.^12^ Particle size affects the adsorption energy.^13^ Smaller particles have faster adsorption kinetics and, hence, have stronger packing efficiency.^14^ When the rate of particle adsorption is faster than the rate of coalescence, the emulsions will be stabilized rather than coalesce.^5^ Due to this stability, PEs have many applications in food, cosmetic, materials, and polymers industries, in oil recovery, wastewater treatment, biomedicine, and in 3D printing. These types of PEs also have huge potential to be used as catalysts in chemical and enzymatic reactions. So far, a comprehensive analysis on the use of PEs as catalysts in food systems is lacking. Many studies focused mainly on PE catalysts in organic synthesis reactions, but not much focus was given on the potential of Pickering emulsions as catalysts in the food industry; for example, in improving the quality of food ingredients by using enzymatic Pickering emulsions. This Review aims to fill this gap. The two types of PE catalysts will be discussed: Pickering-assisted catalysts (PAC) and Pickering interfacial catalysts (PIC) and their applications in foods. Limitations of PAC and PIC that inhibit their widespread use in the food industry are also presented, as well as a discussion of the potential solutions to address these limitations. The future of PE catalysis in foods is also included.

## II Pickering Emulsions as Catalytic Systems

**Figure 1:**
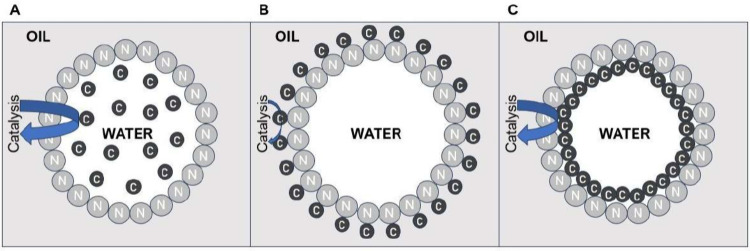
Schematic diagram of water-in-oil Pickering emulsions with (A) Pickering-assisted catalysis, (B) Pickering interfacial catalysis, and (C) Pickering-assisted interfacial catalysis. C = Catalyst. N = Nanoparticle.

When PEs are used as catalytic systems, the catalyst (whether inorganic, organic, or enzymatic) can either be 1) dispersed/dissolved in the oil or water or 2) integrated in the solid particles that act as stabilizers. Pera-Titus et al. introduced the term Pickering-Assisted Catalysis (PAC) to refer to case (1) and Pickering Interfacial Catalysis (PIC) to refer to case (2).^15^ The first example of PIC was reported by Crossley et al.^16^ They performed hydrodeoxygenation of a phenolic compound and hydrogenation and etherification of an aldehyde in W/O PEs using Pd/carbon nanotube-inorganic oxide hybrid nanoparticles as stabilizers.^16^ Nowadays, the oil and water phases have become more varied. Oils can range from vegetable oils and aromatic oils to nonpolar alkanes, while polar solvents can be ethylene glycol and ethyl acetate. Ionic liquids are also becoming increasingly used due to their desirable properties as green solvents.^17^ The difference between PAC and PIC is that, in PAC, the adsorbed solid particles serve only as stabilizers, while in PIC, the solid particles act as both stabilizers and catalysts for the reaction in the emulsion.^18^ In Zhang et al., they showed that, as the droplet size increases, it reaches a certain size where a PAC-type PE starts behaving as a PIC.^19^ The catalysts are then localized around the surface inside the droplet. A general scheme that highlights the difference between PAC, PIC, and an “intermediate” form, Pickering-assisted interfacial catalysis, is shown in Figure 1.

While traditional techniques of catalysis such as single-solvent catalysis are advantageous over PE catalysis when the reactant and the catalyst (or enzyme) are in the same phase, PE catalytic systems are better when the reactants, catalysts, or products have very different solubilities in the oil and water phase. PE catalytic systems offer possibilities of compartmentalization of the reactants and products, easy catalyst recyclability and reusability, protection of the enzymes, and design of multistep catalytic reactions.^20,21^ In addition, there is no need to use solvents for extraction and purification steps.^20^

### A Pickering-Assisted Catalysis (PAC)

Pickering-assisted catalysis (PAC) occurs when the catalysts are located in the liquid phase. Biocatalysis is a rich field of application for PAC. The enzyme (catalyst) is usually located in the aqueous phase and the solid particles that act as stabilizers are in the liquid–liquid interface.^22−27^ PAC finds uses in nonaqueous biocatalysis and multistep reactions.^28^ When enzymes are used, a W/O PE is preferred, since the aqueous phase forms the dispersed phase. The aqueous phase generally comprises salt-buffered aqueous solutions. So far, it has been observed that neither the type of buffer salt nor the pH has an effect on the formation and the stability of the emulsions.^28^ Silica particles are popular stabilizers of W/O emulsions due to their biocompatibility and ease of surface modification, while lipase enzymes dissolved in the aqueous phase are common catalysts for hydrolysis and esterification reactions.^17^

A proof-of-concept study introduced the use of PEs in a PAC reaction.^26^ The authors encapsulated three types of enzymes in PEs using hydrophobized silica nanoparticles as “cages”, which they termed “colloidosomes”. The enzymes were lipase B from *Candida antarctica* (CalB), lipase A from *Candida antarctica* (CalA), and a delicate enzyme, benzaldehyde lyase from *Pseudomonas fluorescens* Biovar I (BAL).^26^ The aqueous phase of the PEs was jellified with a 1.5% agarose gel. The jellification of PEs increased the stability of the vulnerable enzyme BAL and prolonged its operationability. This was one of the earliest studies that addressed the deactivation of delicate enzymes in PE catalysis.

Some novel PAC systems were reported by Yu et al.: the enzyme was dispersed in the continuous aqueous phase of an O/W Pickering emulsion using silica nanoparticles as stabilizers and the PEs were demulsified by bubbling N_2_ at room temperature;^29^ and by Zhang et al.: the PEs were nonaqueous but rather, ionic liquids (IL) of varying hydrophobicity/hydrophilicity were used where the catalyst was in the IL in an IL/O emulsion.^30^

An “intermediate” form of PAC/PIC is Pickering-assisted interfacial catalysis.^31^ It has been proposed that in biocatalytically active PEs, the dissolved enzymes situate themselves at the interface, creating a Pickering-assisted interfacial catalytic system. This is distinct from PAC where the enzymes are found in the dispersed phase and from PIC since the enzymes are not bound to the stabilizing particles but are nevertheless located at the interface.^31^

#### Application in Food Systems

An application of biphasic PAC in foods was reported by Yu et al.^29^ They hydrolyzed olive oil using O/W PEs with silica nanoparticles as the stabilizers and the lipase from *Candida rugosa* loaded in the aqueous phase. The hydrolytic conversion of olive oil reached as high as 91% using the biphasic PE system.^29^ After the reaction, the PEs were demulsified by bubbling N_2_ gas at room temperature. By doing so, the products in the oil phase were separated from the enzyme and silica nanoparticles in the water phase. The enzyme can then be recycled multiple times, which then reduces the costs of the enzymatic reaction especially in the food industry (Table 1). Cases where extra stability of PACs is important are where emulsion sediments to form a fluidized bed-type reactor,^32^ in continuous flow-type reaction columns,^33^ and in multistep reactions catalyzed by different catalysts/enzymes in separate compartments.^34^

**Table 1:** Examples of PE Catalysis Applications with Potential Uses in Foods

Type of Catalysis	Catalyst	Solid particle	Process	Reference
PAC	Lipase	Silica nanoparticle	Hydrolysis of olive oil	(^29^)
PIC	Lipase	Chitosan nanogel using genipin as cross-linking agent	Formation of edible Pickering interfacial catalyst	(^46^)
PIC	Lipase	α-Lactalbumin nanotube	Enhanced release of fatty acids for low-fat cheeses	(^52^)
PIC	Lipase	Magnetic microcrystalline cellulose	Refining of crude rice bran oil	(^53^)
PIC	Lipase	Fe_3_O_4_@SiO_2_ nanoparticle	Deacidification of rice bran oil	(^48^)
PIC	Lipase	Acetylated arachin nanoparticle	Preparation of diacylglycerol (DAG)-rich soybean oil	(^56^)

### B. Pickering Interfacial Catalysis (PIC)

Pickering interfacial catalysis (PIC) is defined as a PE system in which the solid particles also act as the catalysts at the two-phase liquid–liquid interface. The solid particles can be linked to enzymes for an enzymatic reaction, or they can be surface-modified to support an inorganic catalyst.^35−38^

The highest adsorption energy is achieved when the contact angle is 90°. Larger particles require higher adsorption energy than smaller particles with the same contact angle.^5^ To obtain a firm anchoring of the particles at the interface, the contact angle should be close to 90° (amphiphilic).^5^ PE stabilizers with a contact angle approaching 90° produce more stable emulsions due to their high affinity to the interface that separates the continuous and dispersed phases.^39^ In addition, some studies have shown that particles with contact angle = 90° or close to 90° can facilitate the formation of double emulsions due to their ability to adsorb at both O/W and W/O interfaces.^40−42^ Therefore, to achieve workable and efficient PIC systems, the design and fabrication of amphiphilic solid materials are crucial. Although there are solid particles that make good supports and catalysts, they might not be able to form stable emulsions. Hence, modifications of the solid particles to make them amphiphilic are needed. Previously reported PIC systems are intrinsic amphiphilic carbon materials and extrinsic amphiphilic particles like chemistry-grafted solid particles and nanohybrid particles.^39^ The size, shape, concentration, and surface properties of the solid particles also affect the stability and efficiency of the PIC. For example, theoretically, sheet-like particles are more powerful stabilizers of PEs than spherical particles of the same size and composition due to the higher detachment energy of sheet-like particles from the interface.^43^ Meanwhile, higher particle concentration improves the emulsion stability because the particle package layer around the droplets becomes denser and thicker, preventing droplet coalescence and reducing the droplet size.^44^ In summary, the characteristics of the solid particles make a difference in designing PIC systems.

#### Application in Food Systems

PEs have a large specific surface area and due to this property, they have improved catalytic efficiency as interfacial catalytic reactors^39^ (Table 1). PEs can also be demulsified (the oil phase and water phase are separated) by adjusting the pH, temperature, and freeze–thaw cycles. Hence, a rapid separation of catalytic substrates and products can be achieved.^39^ In a study by Xi et al., PEs prepared by phosphorylated zein particles conjugated into the gold nanoparticles can act as catalysts for oil–water interface cascade reactions.^45^ At present, there are few studies of PEs as interfacial catalytic reactors in foods. Most of previous studies used PEs as catalysts, but the particles are not food-grade, and this poses a problem when aiming at developing food systems.^42,46−51^ In one study, where chitosan was used as solid particles and was modified *in situ*, the PEs carrying interfacial lipases still maintained 55% of its initial activity.^46^ Genipin was used as a cross-linking reagent for its toxicological safety.^46^ To separate the products and reactants, the PEs underwent phase inversion. The phase inversion was facilitated by an enzymatic trigger reaction (the system was designed to be a stimulus-responsive “smart” Pickering emulsion) when free fatty acids released from the lipase hydrolysis interacted with the chitosan surface, hence affecting the wettability of the chitosan particles.^46^ In another study, α-lactalbumin nanotubes were used as particles to immobilize the lipase and improve its catalytic activity.^52^ These are a few examples of potential edible Pickering interfacial catalysts.

**Figure 2:**
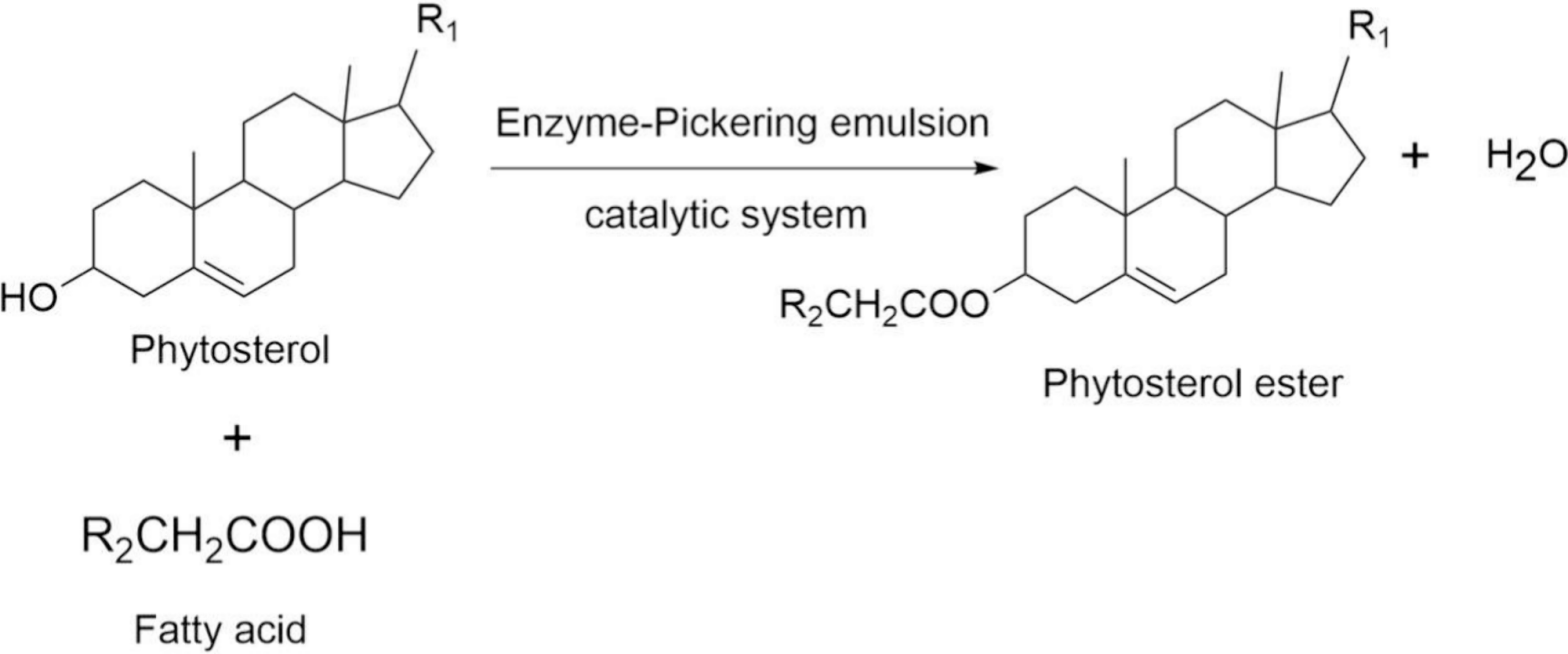
Formation of phytosterol esters from phytosterol and fatty acid facilitated by the Pickering emulsion catalyst.

Another example of potential food application of PIC is in the refining of edible oils; for example, rice bran oil. Until now, the high cost and large environmental impact of refining traditional rice bran oil have been the biggest challenges facing their widespread use. In one study, a lipase-catalyzed hydrolysis reaction of crude rice bran oil was performed using PIC with modified magnetic microcrystalline cellulose (MMC) as solid particles.^53^ The MMCs exhibited a rod-like structure with a contact angle of 84.8°, which indicated the hydrophilic nature of the MMCs. The MMCs were used as the emulsifier. Comparison between the hydrolysis rates of crude rice bran oil using PIC and a biphasic system showed that the lipase-conjugated-PIC had a 92.8% hydrolysis rate while the biphasic system had 58.8%.^53^ The relative activity of the lipase was 71.8% after 10 cycles. In Cheng et al., the FFAs released were envisioned to be used in biodiesel, but their study has proven that it is possible to produce edible oils with high content of desirable fatty acids, depending on the oil profiles, using lipases.^53^ For example, increasing the amount of free medium-chain fatty acids is beneficial since these fatty acids have been shown to possess antiobesity and antidiabetic properties.^54^ Using Fe_3_O_4_@SiO_2_ nanoparticles as stabilizers of PEs, Wang et al. were able to deacidify rice bran oil using lipases.^48^ This system was characterized by a short reaction time (6 h) and recyclability of the lipase (12 reuses with a relative activity of 72%) (Figure 2).^48^

**Figure 3:**
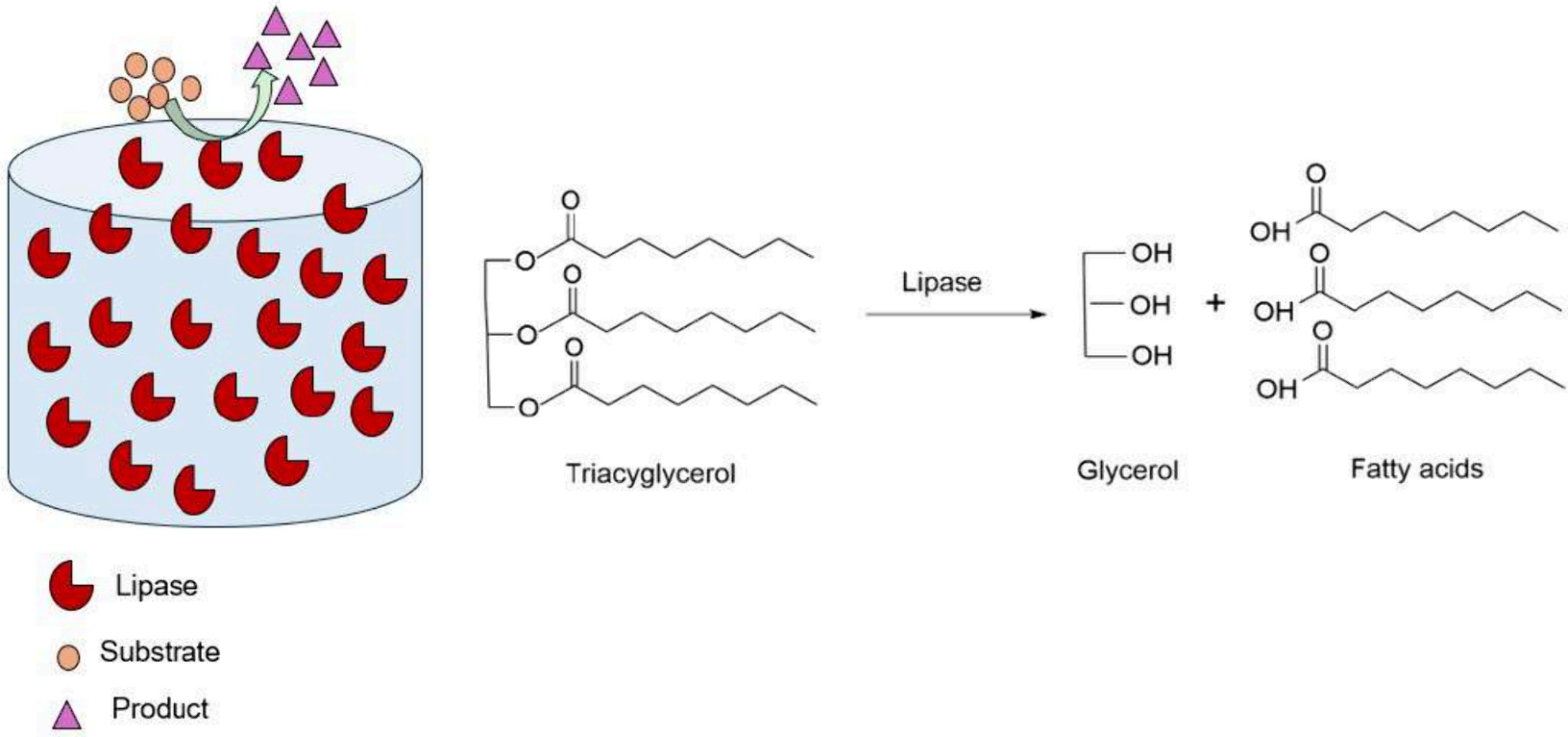
Schematic diagram of a lipase-nanotube-stabilized reaction to transform triacylgerols into fatty acids.

Another application of PIC is in the production of low-fat cheeses. Flavor defects due to insufficient free fatty acid (FFA) flavor release are one of the limiting factors for low-fat cheese consumption. In one study, lipases were immobilized onto α-lactalbumin nanotubes that acted as the solid particles in PEs.^52^ Their aim was to increase the lipase activity and address the flavor defects in low-fat cheeses by lipase hydrolysis. Guan and co-workers found that lipase activity was improved by 68% using lipase nanotubes compared with free lipase.^52^ The affinity of the lipases to their substrates and their catalytic efficiency also increased when the lipases were immobilized to the nanotubes^52^ (Figure 3). When the lipase-nanotube-stabilized milk fat O/W Pickering emulsions were added in low-fat cheese to replace the original milk fat, the amount of FFA released was doubled compared with normal low-fat cheese.^52^ This shows that the flavor of low-fat foods can be enhanced by immobilizing lipases in PIC to improve enzyme activity and increase FFA flavor release.

PEs can be used as catalysts in the production of diacylglycerol (DAG)-rich oils. DAG is a type of oil that has potential to prevent obesity and cerebrovascular diseases.^55^

Acetylated arachin as nanoparticles (AAPs) and as immobilization carriers was used to increase the activity of free lipase.^56^ Acetylation increased the hydrophobicity of the arachin. When used to prepare DAG-rich soybean oil, the PEs with lipase-immobilized AAPs had 2.36× more efficiency than a similar biphasic catalytic system.^56^ This provides a promising way to promote the production of DAG.

## III Limitations as Catalysts in Food Systems

One of the limitations of PE catalysts in both PAC and PIC in food systems is the limited options of food-grade solid particles to stabilize PEs. If they do exist, food-grade solid particles such as polysaccharides, proteins, and inorganic complexes have poor dual wettability, which requires physical and chemical modifications of these materials. In turn, surface modifications can affect the PE stability. It becomes a question of favoring either functionality or stability, and a balance must be achieved for both aspects. In addition, the concentration of solid particles, pH, electrolyte concentration, and volume ratio of oil to water contribute to PE catalytic properties. The recyclability of PE catalysts should also be improved and needs to be considered when designing PE systems. For PAC, one limitation is the deactivation of the enzymes during immobilization within the PE. Many industrially relevant sophisticated enzymes cannot be used in PAC due to their vulnerability to environmental changes.^26^

## IV Proposed Solutions to Address the Limitations

The limitations that prevent widespread use of PE catalysts in food systems are the narrow range of currently available food-grade solid particles as stabilizers, the poor wettability of these particles that requires modifications, their recyclability, and the potential deactivation of the enzymes. One study by Huang et al. addressed the limitation of food-grade stabilizers, recyclability, and stability.^46^ Using chitosan nanogels formed from a strong dispersion of chitosan aggregates as the particle stabilizers in O/W PEs, they were able to enhance the catalytic activity of lipases, the recyclability, and pH stability.^46^ They were also able to design stimulus-responsive PEs that can undergo phase inversion during enzymatic triggers to separate the substrates and the products of the hydrolysis reaction.^46^ Recently, modified starches have also been developed as stabilizers of PEs. Native starches are unstable and highly hydrophilic, and form large particles, resulting in poor emulsifying properties; that is, they cannot be adsorbed properly at the oil–water interface.^57^ Possible modification techniques for starches include acid hydrolysis, nanoprecipitation, alkaline treatment of starch nanocrystals, and chemical reactions to increase the hydrophobicity of the native starches, such as with octenyl succinic anhydride, as reported by Song et al.^58^ Cellulose and its derivatives have been used as stabilizers of the PEs. Increasing the hydrophobicity of the cellulose microparticles appeared to increase the effectiveness of the cellulose in emulsifying oil than that of native cellulose microparticles.^59^ Another group of food-grade stabilizers of PEs is proteins, such as soy, whey, and potato proteins that have been stabilized and either complexed or cross-linked with polysaccharides, phenolic acids, and flavonoids, respectively.^60−62^ The treatments mentioned previously changed the properties of the food-grade stabilizers, particularly their hydrophobicity and hydrophilicity, to increase their effectiveness as stabilizers of PEs.

Modifier-free PEs are possible to synthesize by controlling the concentration of the stabilizer particles, the emulsion size, and the functional particle coverage. By a rational synthetic approach, nano- or microparticles of different morphologies, compositions, surface chemistry, and functionalities can be designed. For example, Zhang et al. produced a modifier-free type of PEs by a promoter-assisted coassembly approach using carbon nanotubes and gold particles.^63^ The functional nanoparticles were assembled with another type of particle that acted as an emulsion stabilizer. This strategy to prepare modifier-free PEs can facilitate their widespread use in catalysis in food production, especially when food-grade particles are employed.

Yu et al. designed W/O PE emulsions as a recyclable microreactor using chitosan-modified ethyl cellulose particles as solid particle emulsifier and lipase as the encapsulated enzyme.^64^ Their study offers solutions to two limitations of PE as a catalysts. Ethyl cellulose and chitosan are food-grade materials, are widespread in nature and are environmentally sustainable. The type of emulsions can also be shifted easily from W/O to W/O/W and O/W as the water fraction increased. In addition, the encapsulated lipase remained stable and could be recycled over 15 times without the loss of activity.^64^

In general, when enzymatic reactions are involved, such as in the case of lipase, “interfacial quality” is the key influencing factor for enzyme bioactivity. Interfacial engineering becomes an effective strategy.^65^ Li et al. designed triblock copolymers of poly(ethylene oxide)-poly(propylene oxide)-poly(ethylene oxide) (PEO-PPO-PEO) to form conventional O/W emulsions.^65^ Depending on the types of lipases and their molecular weights, the adsorption ratio can be altered. In their case, they found that a smaller lipase exhibited a higher interfacial adsorption ratio than a larger lipase.^65^ Following this example in conventional emulsions, the catalytic activity of PEs as catalysts can also be enhanced by the improved design of tailored interfaces or interfacial engineering. For example, in the case of PAC, the enzyme bioactivity depends on the sensitivity of the enzyme to environmental conditions. Wu et al. appeared to circumvent this challenge by immobilizing a delicate enzyme in agarose gel in the aqueous phase.^26^ Different types of gels can be synthesized in the dispersed phase to improve the enzyme stability. The advantages and disadvantages of using PE catalytic systems compared with single-solvent catalysts are summarized in Table 2.^20^

**Table 2:** Advantages and Disadvantages of Single-Solvent and Pickering Emulsion Catalysis^a^

System	Advantages	Disadvantages
Single-solvent catalysis	• Simple setup and operation	• Poor catalyst recyclability
	• Homogeneous distribution of catalyst and efficient mixing with the reactants	• Deactivation of catalyst
	• Often high reaction rates	• Separation of homogeneous catalysts
	• Easy scale-up	• Common use of non-“green” solvents
		• Costly solvent disposal
		• Solvent incompatibility with reactants and products
Pickering emulsion catalysis	• Compartmentalization	• Stabilization of emulsions
	• Catalyst recyclability and reusability	• Limited scalability
	• Protection of enzymatic catalyst	• High energy requirement
	• Possibility to perform multistep catalytic reactions	

a Adapted from ref (20).

## V Outlook

PE catalytic systems can be designed to carry other types of enzymes that perform hydrolytic reactions. Other possible enzymes are proteases and glycosidases. Proteases can be used to reduce protein allergen content, while glycosidases can be used in starch- or fiber-rich food products to produce tailor-made structures and modify their rheological properties. In addition, PE catalysts with modifier-free solid particles as stabilizers can be assembled using the strategy previously discussed (coassembly approach) but using food-grade solid particles. Stimuli-responsive “smart” PE catalysts can also be designed to make the separation of the products, the enzymes, and the solid particles easier by phase inversion or demulsification. The field of PE catalysis in food systems is rich in possibilities since there are many ways to change the chemistries and functionalities of the stabilizing solid particles and the emulsions themselves and various strategies to overcome the limitations previously presented. PE catalysis offers a new avenue to design and produce novel food products with improved physicochemical, sensory, and health properties at reduced costs and in a sustainable manner.

## Data Availability

No data was used for the research described in the article.
